# The effectiveness of oxytetracycline in the treatment of calves with contracted flexor tendons

**DOI:** 10.18849/ve.v7i1.455

**Published:** 2022-03-17

**Authors:** Antonia Leech+

**Keywords:** CONTRACTED FLEXOR TENDONS, OXYTETRACYCLINE, NEONATAL CALVES

## Abstract

PICO question

In neonatal calves with contracted flexor tendons is the use of a 3 day course of oxytetracycline in conjunction with other treatments more effective in returning the hoof to normal full weight bearing on both the toe and heel compared to no oxytetracycline?

Clinical bottom line

Category of research question

Treatment

The number and type of study designs reviewed

Three papers were critically reviewed. One randomised controlled study, one case series and one case study

Strength of evidence

Weak

Outcomes reported

Oxytetracycline as a treatment for contracted flexor tendons in calves was found to be slightly more effective in returning the hoof to normal weight bearing compared to no oxytetracycline. In contrast, oxytetracycline infusions for the treatment of contracted flexor tendons in calves do not have an influence on weight bearing and have no significant clinical effect

Conclusion

There was limited confidence that the estimated effect reported by the studies were close to the true effect, this is due to the studies having a number of limitations as well as the case series / study having limited evidentiary power. There is currently insufficient evidence from the literature to support or reject the use of oxytetracycline in the treatment of contracted flexor tendons in calves. Further studies, with higher strengths of evidence, are required to provide conclusive evidence

## How to apply this evidence in practice

The application of evidence into practice should take into account multiple factors, not limited to: individual clinical expertise, patient’s circumstances and owners’ values, country, location or clinic where you work, the individual case in front of you, the availability of therapies and resources.

Knowledge Summaries are a resource to help reinforce or inform decision making. They do not override the responsibility or judgement of the practitioner to do what is best for the animal in their care.

## The evidence

Following refinement of the initial search and exclusion of unsuitable publications, such as papers that made a singular statement about the use of oxytetracycline in calves, one study was found to be directly applicable to the PICO question (Fazili et al., 2014) and two studies partially applicable (Metzner et al., 2007; and Kumar et al., 2012). Fazili et al. (2014) was a randomised controlled study, but even then, had a large number of limitations, insufficient statistical power and therefore an unclear clinical effect. Whereas the others, which were a case series and case study lacked evidential strength, as well as not directly answering the PICO and therefore were of little value (Metzner et al., 2007; and Kumar et al., 2012). None of the studies were completely comparable due to each having slightly different intervention and outcomes. There is insufficient evidence available to either support or oppose the use of oxytetracycline alongside the use of other treatments of calves with contracted flexor tendons. Further randomised controlled trials would greatly help in providing more reliable proof for this PICO question.

## Summary of the evidence

**Fazili et al. (2014):** 

Population:	Neonatal calves (aged between 1–25 days old) with moderate congenital fetlock knuckling presented to the Teaching Veterinary Complex, Faculty of Veterinary Sciences, Sher-e-Kashmir University of Agricultural Sciences and Technology of Kashmir, Shuhama, Srinagar, Kashmir, India by rural owners for treatment over a period of 3 years. Calves were included if they had moderate bilateral contracted flexor tendons, forelimbs were involved in 12 calves, and in two calves, hindlimbs were affected. Calves were excluded if they had additional deformities. The angle of the deformity of the fetlock joint was measured in degrees. The prevalence was calculated as the proportion of the calves with knuckling among the neonates showing musculoskeletal congenital defects and was calculated as a percentage.
Sample size:	70 new-born calves with congenital external abnormalities were presented to the University. 14 calves of 19 that were presented with knuckling of the distal limb joints. Five of those 19 were discarded due to also having additional abnormalities.
Intervention details:	The calves were restrained without premedication in lateral recumbency and the angle of the affected fetlock joints were measured using a protractor. The calves were either injected with 0.05 mg/kg of xylazine intramuscularly (IM) or 0.1 mg/kg of diazepam intravenously (IV) and were restrained in lateral recumbency. A splint made from wooden or polyvinyl chloride (PVC) pipe was cut into one-third or half semi-tubular shape and applied to the palmar / plantar aspect of the affected limbs as a splint. All calves were given a single dose of 2.0 mg/kg IM tolfenamic acid as an analgesic. The calves were randomly allotted to one of the two equal groups (n=7), Group I and Group II. Group I was given oxytetracycline at 20.0 mg/kg with the calculated dose of oxytetracycline added to 250 ml of normal saline and infused IV daily for 3 consecutive days alongside splints. Group II received no additional medication, just splints. The owners were directed to apply three to four additional layers of bandage after carefully stretching the limb every alternate day and were instructed to remove the splints on day 10 and to encourage the animals to stand and walk for 2 more days before presentation to the hospital. The calves were evaluated after 12 days and then 20 days, following institution of the treatment.
Study design:	Prospective randomised control study.
Outcome studied:	An objective assessment on the prevalence of contracted tendons as one of the common congenital musculoskeletal abnormalities affecting neonatal dairy calves. An objective assessment on the effect of using a 3 day course of oxytetracycline at a dose of 20.0 mg/kg in reducing the angle of the contracted fetlock deformities alongside the use of a splint.
Main Findings (relevant to PICO question)	9/12 (75.0%) of limbs were cured in 12 days when treated with oxytetracycline compared to 7/10 (70.0%) of limbs cured with no additional treatment. 10/12 (83.3%) of limbs were cured in 20 days when treated with oxytetracycline compared to 8/10 (80.0%) of limbs cured when not using oxytetracycline. Use of oxytetracycline at a low-toxicity dose (20 mg/kg IV) daily for 3 consecutive days has a small additional beneficial effect in managing moderate fetlock knuckling.
Limitations:	19 calves were found to have knuckling of the distal limb joints however only 14 were used in the study. It does not clearly state the reasons why the five were excluded from the study, or whether forelimbs knuckling responded differently to treatment compared to hindlimb knuckling. Small sample size of 14 calves. There was a wide age variety of the calves varying from a day to 25 days old. It was not mentioned as to how age could have affected response to treatment. Either xylazine or diazepam was used as a sedative with some muscle relaxant effect, however the difference in said effect, the number of calves treated with either and the reason for deciding between the two were not stated. Consequently, adding bias and potentially affecting the stretching of the contracted tendons and therefore the results. Both wooden (even though they were stated to have more complications) and PVC splints were used in the treatment of the calves. However, there was no definition of how many of each and how they were distributed over the two sample groups and whether these were chosen randomly which could be a source of bias. As the purpose of the study was to measure the effect of oxytetracycline, the other variables, such as splints should have been controlled and therefore kept as the same materials. As a result, it is difficult to establish whether the improvement was due to the use of oxytetracycline or the type of splint used. The randomisation technique for splitting between the two sample groups was not stated. With regards to monitoring and treating the calves with splints, different persons were doing this and therefore adding subjective bias. There was no regulation in the number of additional bandages used and the amount of stretching the limbs were succumbed to, as well as the amount of standing and walking the calves had for the 2 days prior to presentation at the hospital. The fetlock angles were compared between and within the groups at either 12 days and 20 days following treatment. The difference in evaluating the calves at different time intervals is not discussed nor differentiated between which calves were seen and when. Three calves were lost (one due to an improperly maintained splint, one lost to follow-up and one due to pressure sores) and therefore made the sample size even smaller. It concluded that the limbs of 9/12 (75.0%) in group I after 12 days had resolved completely however it did not define ‘resolved’ in terms of fetlock angles. A paper is referenced, which measures the angles of joints proximal to the fetlock of calves. It states that this study in question is the first time that fetlock joint angles in calves have been analysed and gives no reference of what they deemed as a ‘normal’ fetlock angle and how they came to that decision. Of the calves not completely resolved, they were then treated for a further week and one fully recovered. It is unclear as to whether this calf was included in the overall statistic of successful treatment after being exposed to a difference in the variable of time. Two of the calves in Group I were left with significant angle deformities upon removal of the splint however, even though they showed weight-bearing they were still lame and showed mild bending of the fetlock and it was not defined as whether they were included as successful. The study period is not defined. Despite the known nephrotoxic effect of oxytetracycline, the kidney function tests could not be conducted, and therefore the benefit risk of its use in knuckling calves still needs to be confirmed. Does not involve any description on how the results were statistically analysed.

**Metzner et al. (2007):** 

Population:	Calves up to the age of 4 weeks with contracted flexor tendons that could stand for less than 1 minute on the tips of their claws without support. The number of limbs of each calf affected were not stated.
Sample size:	10 calves.
Intervention details:	Calves were selected if they could stand for less than 1 minute on the tips of their claws without support. Calves with clinical signs of arthritis were excluded. All 10 calves were included in the results. Goniometry, in degrees, measured the angle of the fetlock joint. The time was measured in seconds and recorded how long the calf could stand for without knuckling and without support. During goniometry, the calves were placed in lateral recumbency, and the upper forelimb was fixed horizontally, at a right angle to the longitudinal axis of the body. Marks were made using permanent marker on the lateral aspect of the limbs at the height of the carpometacarpal and metacarpophalangeal joints as well as the coronet. The fetlock joint was then extended with a constant force of five kiloponds (kP) controlled by the use of a spring balance. As a counterforce, a tape was applied around the dorsal aspect of the fetlock joint, by which the foot was pulled back. Goniometry was carried out on both affected forelimbs and the mean of both measurements recorded. The period the animal could stand on its claws without support was recorded daily. Oxytetracycline was infused at a dose of 60 mg/kg intravenously (IV) in 1.01 ml of physiological saline and administered to the calves for 3 consecutive days by the use of a catheter placed in the jugular vein. Goniometry was performed one day before oxytetracycline administration (day 0); immediately before, 30, 60 and 180 minutes after infusions on days 1–3 and 24 hours after the last infusion (day 4). Following the study all calves were treated by other orthopaedic procedures such as immobilisation in a cast or surgical treatment.
Study design:	Case series.
Outcome studied:	An objective assessment on the effect of using a 3 day course of using oxytetracycline at a dose of 60 mg/kg IV as the sole treatment on the goniometry of fetlock joint angulations as well as the time the animal could stand on its claws without support on calves with contracted flexor tendons.
Main Findings (relevant to PICO question)	The median differences in angulation between day 0 (before infusions) and days 1–4 were +0.3°, +6.3°, +9.5° and +7.5°, respectively, indicating a slight extension. However, the differences on days 3 and 4 were the only values statistically significant. The median differences in results between goniometry measurements taken immediately before the oxytetracycline infusion and at 30, 60 and 180 minutes post infusion were -0.3°, -1.0° and 2.0°, respectively (negative values indicate an increased flexion). The median standing time increased from 3.8 seconds on days 0 to 3.8 seconds on day 1; 6.8 seconds on day 2; 9.3 seconds on days 3; but decreased to 3.8 seconds on day 4. For statistical evaluation the Wilcoxon test for paired observations was used at a 5% level of significance and all differences to day 0 were statistically significant with p>0.05. Overall the results suggest that oxytetracycline infusions do not have an influence on the results of goniometry or the time standing without knuckling and therefore has no significant clinical effect on contracted flexor tendons in calves.
Limitations:	There is no in detail exclusion criteria mentioned, just that calves with clinical signs of arthritis were excluded. It does not mention whether any other musculoskeletal defects were excluded which may have been confounding and not have been considered during analysis. Does not state the breed(s) that were assessed and therefore could have had varying results. Does not state where the calves were located, whether they were from the same or different farms and therefore exposed to different variables such as management factors. Small sample size so therefore reduced statistical power. There is no control group to compare. There is no randomisation and no reliable system for selecting discussed and so there is room for potential selection bias. Short duration of the experiment and follow-up limited to 24 hours after the last infusion. There is no discussion as to whether any animals were lost to follow-up and whether all the animal were properly accounted for. Goniometry was performed at immediately before, and 30, 60 and 180 minutes after infusions. However, there is no breakdown of results on each day, just a median of the total days and it is therefore hard to analyse the immediate benefit after each daily dose and whether there was any improvement on this throughout the duration of the experiment. There is no evidence to describe as to how they defined their cure criteria and the scientific reasoning behind this.

**Kumar et al. (2012):** 

Population:	A Karan Fries crossbred calf with both hind contracted flexor tendons.
Sample size:	One calf.
Intervention details:	The calf was restrained and the fetlock joints were fixed in an extended position with plaster of Paris bandage. Oxytetracycline was administered intramuscularly (IM) at 1 ml / 10 kg body weight (which equates to 20 mg/kg) for 5 days. Calf was supplemented with oral minerals and multivitamins for a period of 2 weeks. Calf was kept in plaster bandage for 15 days and then removed. The aim of the treatment was defined as the calf being able to ‘walk normally’.
Study design:	Case study.
Outcome studied:	An assessment of the use of treating contracted flexor tendons with plaster casting and oxytetracycline at a dose of 1 ml / 10 kg (20 mg/kg) IM for 5 days.
Main Findings (relevant to PICO question)	The calf treated with plaster casting and oxytetracycline was able to walk normally without flexion of hind fetlocks after 15 days.
Limitations:	The aim of the treatment ‘walking normally’ was not defined and so could be subjective. There is no clarification of what the supplemented oral minerals and multivitamins were and whether they had an effect on the results. The effect of oxytetracycline is not explained and there is no explanation as to why it has no direct effect.

## Appraisal, application and reflection

Congenital contracted flexor tendons, most commonly of the metacarpophalangeal or metatarsophalangeal joints within 1–2 weeks of birth, are a common defect in numerous breeds of cattle. Possible aetiologies include in utero nutrition, malposition and the foetus being too large relative to the dam. Commonly the calves with flexural deformities may be unable to nurse and therefore at risk of failure of passive transfer of immunity and chronic deformities may lead to skin ulceration and subsequently septic arthritis; both of which, including the aetiology, may be factors influencing treatment success (Fazili et al., 2014).

Anderson et al. (2008) classified flexural deformities as mild (if the calves are able to walk on their feet but the heels do not contact the ground), moderate (if the dorsal aspect of the hoof breaks over a vertical plane perpendicular to the ground), or severe (if the affected animals are forced to walk on the dorsal aspect of the pastern, fetlock, or carpus). They also indicated that treatment for moderate cases, such as the cases found in the literature search, are routinely treated by using a bandage, splint, or cast and by providing analgesia using a non-steroidal anti-inflammatory drug. Several authors have reported that the same disease in foals can be influenced by infusions of high doses of oxytetracycline at 50–70 mg/kg (Lokai, 1992; and Madison, 1994). However, Anderson et al. (2008) reported that high doses of oxytetracycline are contraindicated in calves because of the risk of inducing renal failure, but a lower dose has been used with some success. However, a scientifically sound validation is lacking for the use of oxytetracycline in conjunction with plaster casting in the treatment of calves with contracted flexor tendons.

The literature search performed by the author found one paper which addressed and two papers partially addressing the PICO question. Two papers were shown to measure the effects of oxytetracycline alongside plaster casting or splints (Fazili et al., 2014; and Kumar et al., 2012) and the other evaluated the effect of oxytetracycline as a single treatment and then stated that other orthopaedic procedures such as casting were used after the study ended (Metzner et al., 2007). In the randomised control study (Fazili et al., 2014), a sample size calculation was lacking as well as in the other studies statistical analysis of data was poorly reported, thus hindering interpretation of their significance. The studies were flawed due to their small sample size, lack of randomisation and detail on potential exposure to variable management conditions, thus bias cannot be ignored when reviewing the studies.

The paper by Fazili et al. (2014) concluded that bilateral moderate fetlock knuckling in neonatal dairy calves can be managed satisfactorily with early application of splints and that the supplementary use of oxytetracycline, at 20 mg/kg, had only a marginally beneficial effect. The study compared the treatments using splints with and without oxytetracycline. The treatment group with oxytetracycline found the left and right fetlock angles to reduce from 50.57° ± 4.20° to 4.00° ± 2.27° and 48.71° ± 2.37° to 5.33° ± 3.03° respectively. The treatment group, with just splinting, found the angles of the left and right fetlocks to also reduce from 50.86° ± 2.94° to 4.20° ± 2.70° and from 48.71° ± 3.14° to 6.80° ± 3.34° respectively. Reduction in the angles of both treatment types, when compared to the pre-treatment values, showed high statistical significance (p<0.01). Comparing the values between the two groups, the splint only group showed to be slightly more beneficial in reducing the left fetlock angle by 46.66° ± 3.14° compared to a reduction of 46.57° ± 2.27° in the oxytetracycline group, so a difference of -0.09° ± 1.07°. Whereas, with the right fetlock, the oxytetracycline group was more beneficial in reducing the angle by 43.38° ± 3.03° compared to 41.91° ± 3.34° in the splint only group, a difference of 1.47° ± 0.31°. As splinting only was shown to be more effective in reducing the left fetlock angle and splinting with the use of oxytetracycline was more effective in reducing the right fetlock angle, it can be argued that the addition of oxytetracycline was not significant in the overall effect of reducing the fetlock angles in calves with contracted tendons, especially as there was no control group with just the use of oxytetracycline as a treatment group to compare against.

Fazili et al. (2014) was the first to record and analyse fetlock joint angles in calves with contracted flexor tendons. However, as the normal angles of the fetlock joints in calves were not defined within the study, the significance of the impact of either treatment in resolving the clinical condition can be questioned, as there is no defined normal reference to allow treatment success to be substantiated. For instance, Şirin et al. (2014) measured joints proximal to the fetlock in calves and found there to be a difference in angles between forelimb and hindlimbs, such that what could potentially be seen as normal for a forelimb, could also be classed as an abnormal angle for a hindlimb. Along with this concern, there are a number of other potential limitations of the trial design and reporting of results within the paper.

There was no definition as to how the two sample groups were randomised. The main variable measured was the use of oxytetracycline, whereas the use of different types of splints (wooden or PVC) were not controlled, and nor was there equable physiotherapy given, these variables could have affected the results thus making them unreliable. Compounding this concern, is a lack of clarification as to which groups, and how many of each, were treated using the PCV or the wooden splints, especially as the PCV splints were described as having fewer complications. The study also did not include a non-treatment group and therefore a conclusion that any intervention is of value cannot be stated with certainty. The study also lacked description on how the results were statistically analysed, as well as limited standardised treatment and monitoring. This lack of objectivity in the study design, along with a small sample size and the marginal difference in outcomes between the two treatment groups, mean that conclusions must be interpreted with caution.

In contrast, Metzner et al. (2007), which solely measured the effect of oxytetracycline infusions and not alongside additional treatments, found it to not influence the results of goniometry and therefore weight bearing. There is, however, no detailed exclusion criteria mentioned and so the possibility of confounding effects of concurrent disease meant that conclusions must be interpreted with caution. The study also did not define whether one or multiple limbs were affected, as the number of limbs with contracted flexor tendons would affect the amount of weight bearing on each limb and potentially the effect of treatment and so this needed to be defined in the study. The lack of control group to compare the use of oxytetracycline against no treatment, as well as the many other limitations such as no randomisation, no definition of cure criteria and lack of follow-up or statistical analysis, meant that a large amount of bias could be present and so the results of this study were difficult to interpret.

The study also used a high dose of oxytetracycline at 60 mg/kg instead of the therapeutic dose of 20 mg/kg which was indicated as the maximum dose in the data sheet (VMD, 2020). Compared to the other two papers (Fazili et al. 2014; and Kumar et al., 2012), this is an exceptionally high dose and therefore the difference in dosing makes comparison of its findings more difficult as the other studies use oxytetracycline at repeated doses of low toxicity at 20 mg/kg. The use of oxytetracycline at triple the data sheet’s maximum dose can also be seen as inappropriate and is contraindicated for its use in clinical practice due to its known renal toxicity in calves (Anderson et al. 2008).

Although the study by Kumar et al. (2012) does not directly address the PICO question, it was one of the few studies found to use oxytetracycline in its treatment protocol for contracted flexor tendons and to use weight bearing as a criterion for successful treatment. However, the study used a low dose of oxytetracycline at 1 mg/kg for 5 days; this is off-license, as it is only licensed for use in cattle at a dose rate of 3–10 mg/kg at 24 hour intervals for 3–5 consecutive days (VMD, 2020). The calf was found to walk normally without flexion of the hind fetlocks after 15 days, although no direct effect was attributed to the use of oxytetracycline. There was no explanation for this conclusion. Furthermore, there were no details of controlled variables nor did it state which supplemented oral minerals and multivitamins were used and whether these and concurrent diseases could be confounding factors. Ultimately, as this was a case study of a single calf, the outcome derived had limited evidentiary value in determining treatment effect.

In summary, the findings of this review suggest that there is little significant evidence that the use of oxytetracycline in the treatment of contracted flexor tendons in conjunction with other treatments has a positive or negative effect. With regards to the latter two studies being a case series and case study, the available evidence is weak, meaning they have limited evidentiary power, have a high likelihood of bias and cannot reliably prove the effect of treatment. Although, the randomised controlled study suggested that the use of oxytetracycline has a small significant effect, due to the large amount of limitations, its significance and evidentiary value must be questioned, especially as this review found it to be more effective in only one fetlock and not consistently with both. Undoubtedly, further randomised controlled trials with less limitations are needed to provide better evidence-based information. But until then, clinicians must use their own clinical judgement from available evidence and experience to decide on the necessary treatment for their patient.

## Methodology Section

**Search Strategy:** 

Databases searched and dates covered:	CAB Abstracts on OVID Platform 1973–2022 PubMed accessed via the NCBI website 1910–2022 Web of Science 1945–2022
Search strategy:	CAB Abstracts, PubMed and Web of Science: (Calve* OR Calf) AND (Contracted flexor tendon* OR Congenital knuckling OR fetlock knuckling OR Tendon contract*) AND (Oxytetracycline OR Tetracycline*)
Dates searches performed:	20 Jan 2022

**Exclusion / Inclusion Criteria:** 

Exclusion:	Reviews of available treatments. Book chapters. Non-English language. Papers that could not be accessed by the author or university library. Papers that did not answer the PICO. Papers covering the use of oxytetracycline for treating contracted flexor tendons in other species.
Inclusion:	Studies that answer or relate the PICO question. English language. Accessible by the author or university library.

**Search Outcome:** 

Database	Number of results	Excluded – Not relevant to PICO	Excluded – Not available in the English language	Excluded – Not possible to access study	Total relevant papers
CAB Abstracts	1	0	0	0	1
PubMed	2	0	0	0	2
Web of Science	5	2	1	1	1
Total relevant papers when duplicates removed					3

## Conflict of Interest

The author declares no conflicts of interest.

## Disclaimer

Knowledge Summaries are a peer-reviewed article type which aims to answer a clinical question based on the best available current evidence. It does not override the responsibility of the practitioner. Informed decisions should be made by considering such factors as individual clinical expertise and judgement along with patient’s circumstances and owners’ values. Knowledge Summaries are a resource to help inform and any opinions expressed within the Knowledge Summaries are the author’s own and do not necessarily reflect the view of the RCVS Knowledge. Authors are responsible for the accuracy of the content. While the Editor and Publisher believe that all content herein are in accord with current recommendations and practice at the time of publication, they accept no legal responsibility for any errors or omissions, and make no warranty, express or implied, with respect to material contained within. For further information please refer to our Terms of Use.
